# MPS Content-Dependent Tuning of Optical Properties of MPS-Capped CdS Quantum Dot Thin Films

**DOI:** 10.3390/nano16150900

**Published:** 2026-07-23

**Authors:** Kenan Koç, Anıl Kar, Mustafa Okutan

**Affiliations:** Department of Physics, Faculty of Science and Letters, Yildiz Technical University, Esenler, Istanbul 34210, Türkiye

**Keywords:** CdS quantum dots, (3-mercaptopropyl)trimethoxysilane, thin films, optical properties, Urbach energy

## Abstract

The precise control of optical and dielectric properties of semiconductor quantum-dot-based thin films remains a critical challenge for advanced optoelectronic applications. In this respect, (3-mercaptopropyl)trimethoxysilane (MPS)-capped CdS quantum dot thin films were prepared with MPS:Cd molar ratios of 0.25, 0.50, 1.00, and 2.00 to investigate the role of MPS content in tuning their optical and dielectric properties. The colloidal quantum dot solutions were deposited onto glass substrates by spin coating and subsequently annealed in air at 350 °C. Optical analyses showed that increasing MPS content enhanced film transmittance, whereas the refractive index, extinction coefficient, dielectric loss tangent, and optical conductivity decreased. At 550 nm, the refractive index decreased from 2.12 to 1.61, while the optical conductivity decreased from 6861 to 644 S m^−1^ as the MPS ratio increased from 0.25 to 2.00. The Urbach energy exhibited a non-monotonic variation, reaching a maximum value of 415 meV at MPS:Cd = 0.50 and a minimum value of 193 meV at MPS:Cd = 2.00. Wemple–DiDomenico analysis revealed that the single-oscillator energy increased systematically with increasing MPS ratio, and the $E_{0}/E_{1S-1S}$ ratios remained within a narrow range of 1.32–1.46 throughout the MPS:Cd series. The analysis further revealed that the dispersion energy, static refractive index, infinite-wavelength dielectric constant, and linear susceptibility decreased with increasing MPS molar ratio. The estimated third-order nonlinear optical susceptibility and nonlinear refractive index also decreased with MPS content.

## 1. Introduction

Semiconductor quantum dots have attracted considerable interest due to their size-dependent optical and electronic properties [1,2]. Quantum confinement effects emerge when the dimensions of a semiconductor are reduced to small nanoscale dimensions and become comparable to its exciton Bohr radius [3]. This enables the optical absorption, emission, and electronic response of quantum dots to be tuned by controlling their size, composition, and surface chemistry [1,2]. Among II–VI semiconductor nanomaterials, CdS quantum dots are of particular interest for photonic and optoelectronic applications because of their tunable optical response and strong interaction with light [4].

Colloidal synthesis is a widely used approach for preparing semiconductor quantum dots with controlled size and surface properties [2]. In this method, the choice of capping agent is critical because surface ligands influence nucleation, growth, aggregation behaviour, colloidal stability, and surface passivation [2,5]. Therefore, an appropriate ligand can act not only as a stabilizing molecule during nanoparticle synthesis but also as a functional component that determines the final optical quality of the resulting thin films [5]. In this context, (3-mercaptopropyl)trimethoxysilane (MPS) is a particularly attractive ligand for CdS quantum dots because it combines surface-binding and matrix-forming functionalities within a single molecule. Its thiol group can interact strongly with the surface of II–VI semiconductor quantum dots, thereby supporting nanoparticle formation, surface stabilization, and passivation [6,7]. Previous studies have shown that MPS enables the synthesis of luminescent CdS and ZnS quantum dots with effective size control [6,7], while MPS-capped CdS quantum dots have also been incorporated into thin-film matrices for photonic applications [8]. Beyond its role as a capping agent, MPS is highly useful for thin-film fabrication because its trimethoxysilane group promotes interaction with oxide surfaces and generates a SiO_2_ network through hydrolysis and condensation reactions [9,10,11,12,13,14].

For optical and optoelectronic applications, thin films must simultaneously exhibit high structural quality, suitable transparency, and precisely controlled optical constants [15,16]. Achieving all these requirements in a single material is often difficult. A practical solution is to fabricate composite or hybrid films by combining multiple components, thereby enabling the control of refractive index, thickness, transparency, and optical loss [15,16]. Previous studies on CdS-containing composite films have shown that their optical and dielectric properties can be tuned by varying the matrix type and composition [17,18,19,20]. In this respect, MPS-capped CdS quantum dot thin films provide a promising platform, since the MPS ligand regulates CdS quantum dot growth while also contributing to the formation of an MPS-derived silica-based film matrix [14]. Therefore, a systematic analysis of the MPS-content-dependent optical and dielectric properties of these thin films is important for understanding their potential for optoelectronic applications.

The influence of the MPS:Cd molar ratio on the structural properties and growth kinetics of MPS-capped CdS quantum dots was systematically investigated in a previous study by Koç [14]. Building on that work, the present study focuses on the optical, dielectric and nonlinear optical properties of the thin films as a function of the MPS:Cd molar ratio. For this purpose, MPS-capped CdS quantum dot thin films were prepared with different MPS:Cd molar ratios. By analysing transmittance-derived optical constants together with the Urbach energy and Wemple–DiDomenico parameters, this study provides a systematic evaluation of MPS-content-dependent tuning of optical properties of MPS-capped CdS quantum dot thin films.

## 2. Experimental Procedure

MPS-capped CdS colloidal quantum dots were synthesized following the previously reported procedure [8,14]. Briefly, cadmium acetate dihydrate and thioacetamide were used as cadmium and sulfur precursors, respectively, at a Cd:S molar ratio of 1:1. MPS was used as the capping agent. Cadmium acetate and thioacetamide were separately dissolved in methanol, and MPS was then added to the cadmium precursor solution at MPS:Cd molar ratios of 0.25, 0.50, 1.00, and 2.00. All chemicals were purchased from Sigma-Aldrich (St. Louis, MO, USA). The cadmium and sulfur precursor solutions were subsequently mixed and stirred at 60 °C for 10 min under a nitrogen atmosphere to obtain MPS-capped CdS colloidal quantum dots.

**Figure 1:**
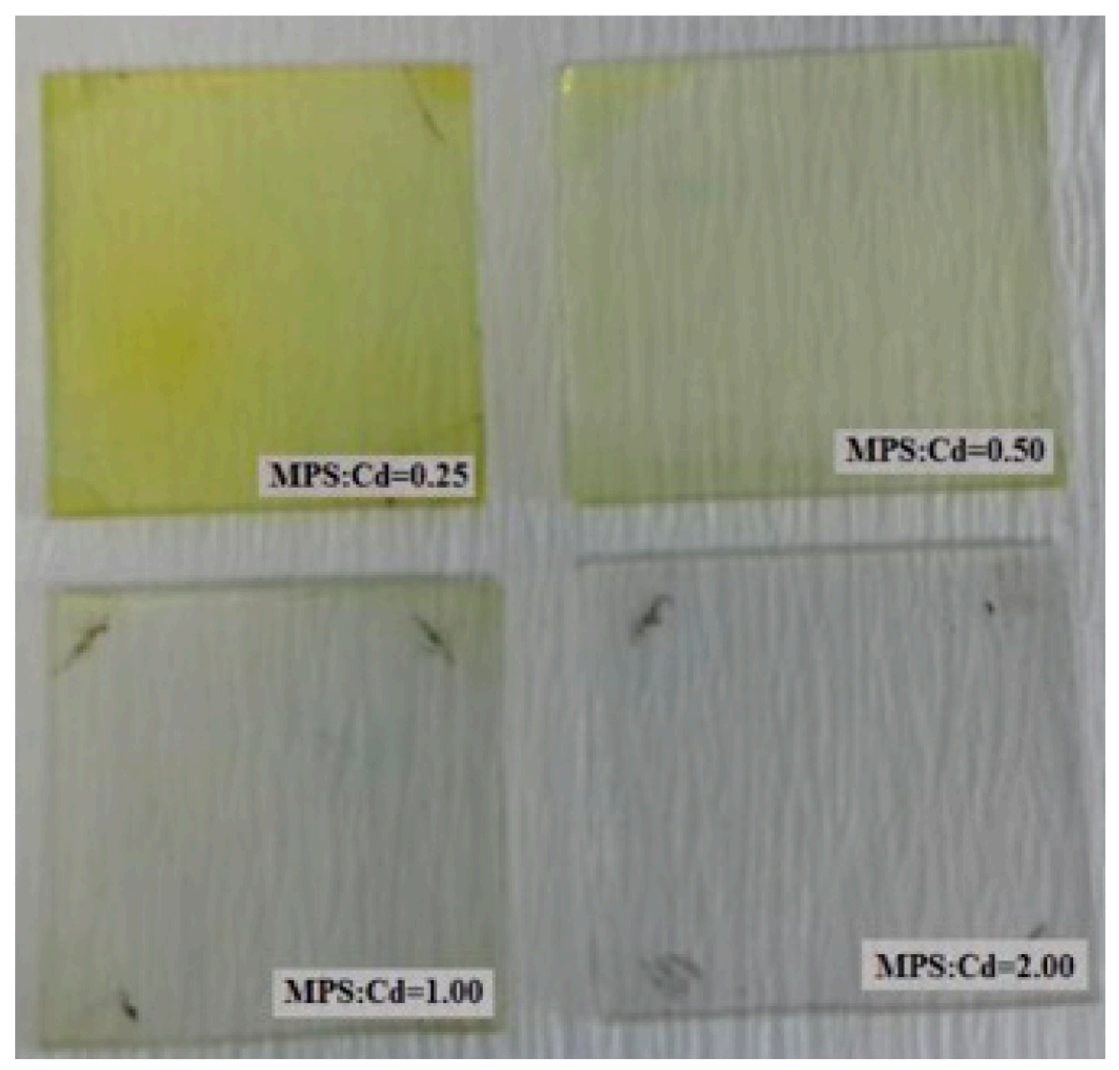
Photographs of thin films prepared from MPS-capped CdS quantum dots with different MPS:Cd molar ratios.

The colloidal solutions were deposited onto Corning 2947 glass substrates (Corning Inc., Corning, NY, USA) by spin coating at 2000 rpm for 20 s. The as-deposited films were dried at 60 °C for 10 min and subsequently annealed in air at 350 °C for 15 min. Photographs of the films prepared with different MPS:Cd molar ratios are shown in Figure 1. The film with an MPS:Cd molar ratio of 0.25 exhibits the most pronounced characteristic yellow coloration associated with CdS, indicating a relatively higher CdS contribution. With increasing MPS content, the films become progressively more transparent, which is consistent with the increasing contribution of the MPS-derived SiO_2_ network and the corresponding reduction in CdS content.

Energy-dispersive X-ray spectroscopy (EDX) analyses were performed using a JEOL JSM-5600LV scanning electron microscope (JEOL Ltd., Tokyo, Japan) equipped with an EDX detector. Optical transmittance spectra were recorded using an NKD-7000V spectrophotometer (Aquila Instruments Ltd., Cambridge, UK) with s- and p-polarized light at an incident angle of 30°. The film thicknesses, refractive indices, and extinction coefficients were determined by fitting the measured transmittance spectra to the Drude–Lorentz model using Pro-Optix™ software, version 4.3 (Aquila Instruments Ltd., Cambridge, UK). A high-resolution transmission electron microscopy (HRTEM) image of the unannealed MPS-capped CdS quantum dot powder sample prepared at an MPS:Cd molar ratio of 0.25 was obtained using a JEOL JEM-2100 microscope (JEOL Ltd., Tokyo, Japan) operated at an accelerating voltage of 200 kV.

## 3. Results and Discussion

**Figure 2:**
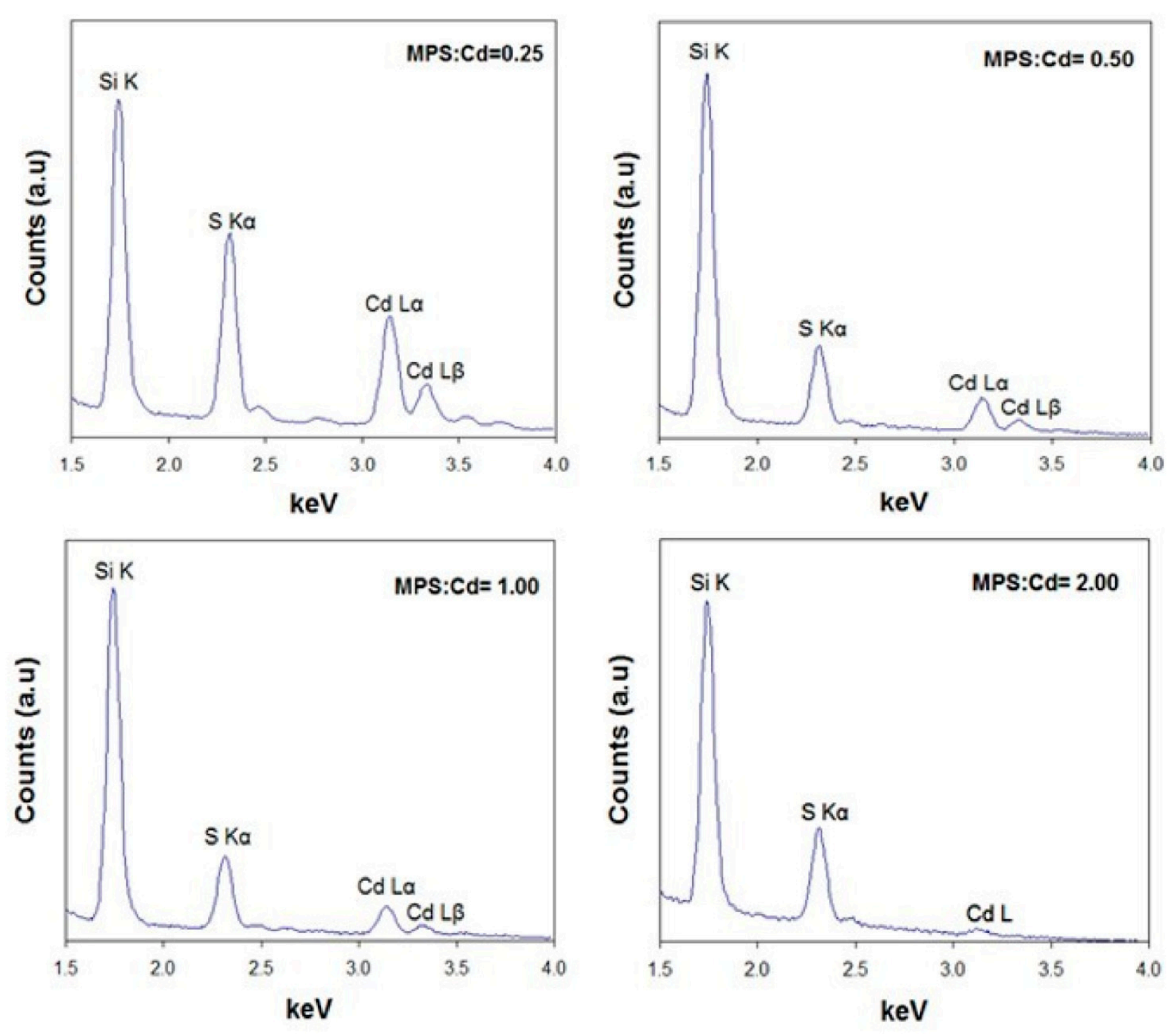
EDX spectra of MPS-capped CdS quantum dot thin films prepared with different MPS:Cd molar ratios.

The EDX results of the MPS-capped CdS nanoparticle thin films with different MPS:Cd molar ratios are presented in Figure 2. As seen from the spectra, the characteristic Si, S, and Cd peaks are clearly detected in all samples, indicating the presence of MPS-capped CdS nanoparticles in each film. The Cd-related peaks originate from the CdS nanoparticles, while the S peaks can be associated with both CdS and the thiol groups of MPS. The Si peaks are consistent with the presence of the MPS-containing matrix; however, considering the thin-film nature of the samples deposited on glass substrates, a possible contribution from the glass substrate cannot be completely excluded. As the MPS:Cd ratio increases, a decrease in the Cd peak intensity is expected due to the reduced relative CdS content in the films. Although the increase in MPS content leads to the expected decrease in Cd contribution, no significant decrease is observed in the S peak intensity. This behaviour can be explained by the competing effects of the decreasing CdS content and the increasing sulfur contribution from the thiol (–SH) groups present in the MPS molecules. Taken together, the EDX results indicate the successful incorporation of MPS-capped CdS nanoparticles into the thin-film structure with varying MPS:Cd ratios. These EDX findings are also in good agreement with the present author’s previously reported X-ray diffraction (XRD) and Fourier-transform infrared spectroscopy (FTIR) results [14], where XRD confirmed the presence of CdS QDs and FTIR evidenced the formation of an MPS-derived SiO_2_ network due to the trimethoxysilane groups of MPS.

**Figure 3:**
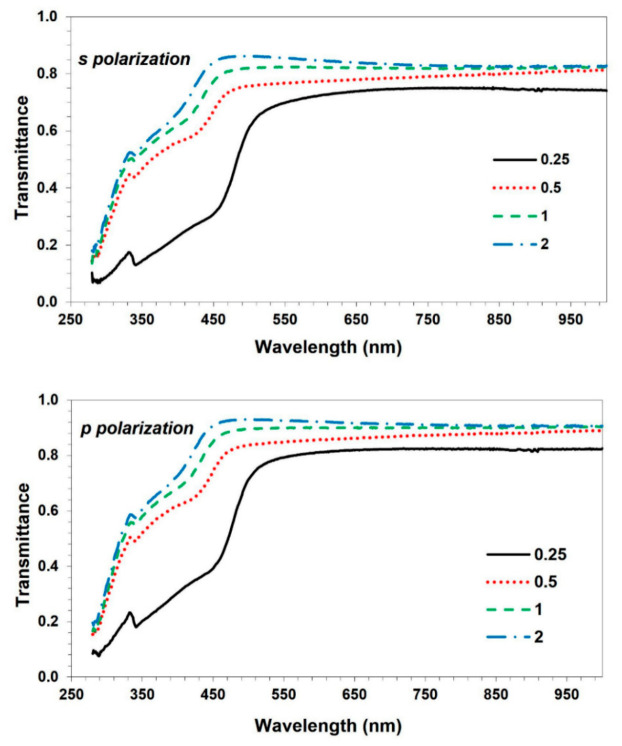
Wavelength-dependent transmittance spectra of MPS-capped CdS quantum dot thin films prepared with different MPS:Cd molar ratios under s- and p-polarized light.

The optical transmittance spectra of the films were recorded using an NKD optical spectrometer. The wavelength-dependent transmittance spectra of the films are presented in Figure 3 for different MPS:Cd molar ratios under s- and p-polarized light. A comparison of the spectra shows that the transmittance under p-polarized light is higher than that under s-polarized light. This difference may be mainly related to the polarization-dependent Fresnel reflection behaviour of s- and p-polarized light at the film interfaces under oblique incidence, rather than being a direct indication of intrinsic optical anisotropy in the films [21]. Furthermore, the transmittance of the films increases with increasing MPS content. The spectra also indicate that, as the MPS:Cd molar ratio increases, the shoulder region shifts toward shorter wavelengths, corresponding to higher photon energies, which is consistent with the quantum confinement effect. For the films prepared with MPS:Cd molar ratios of 0.25, 0.50, 1.00, and 2.00, the transmittance values at 550 nm under p-polarized light are 0.79, 0.85, 0.90, and 0.93, respectively. The corresponding values under s-polarized light are 0.70, 0.77, 0.82, and 0.86, respectively.

**Figure 4:**
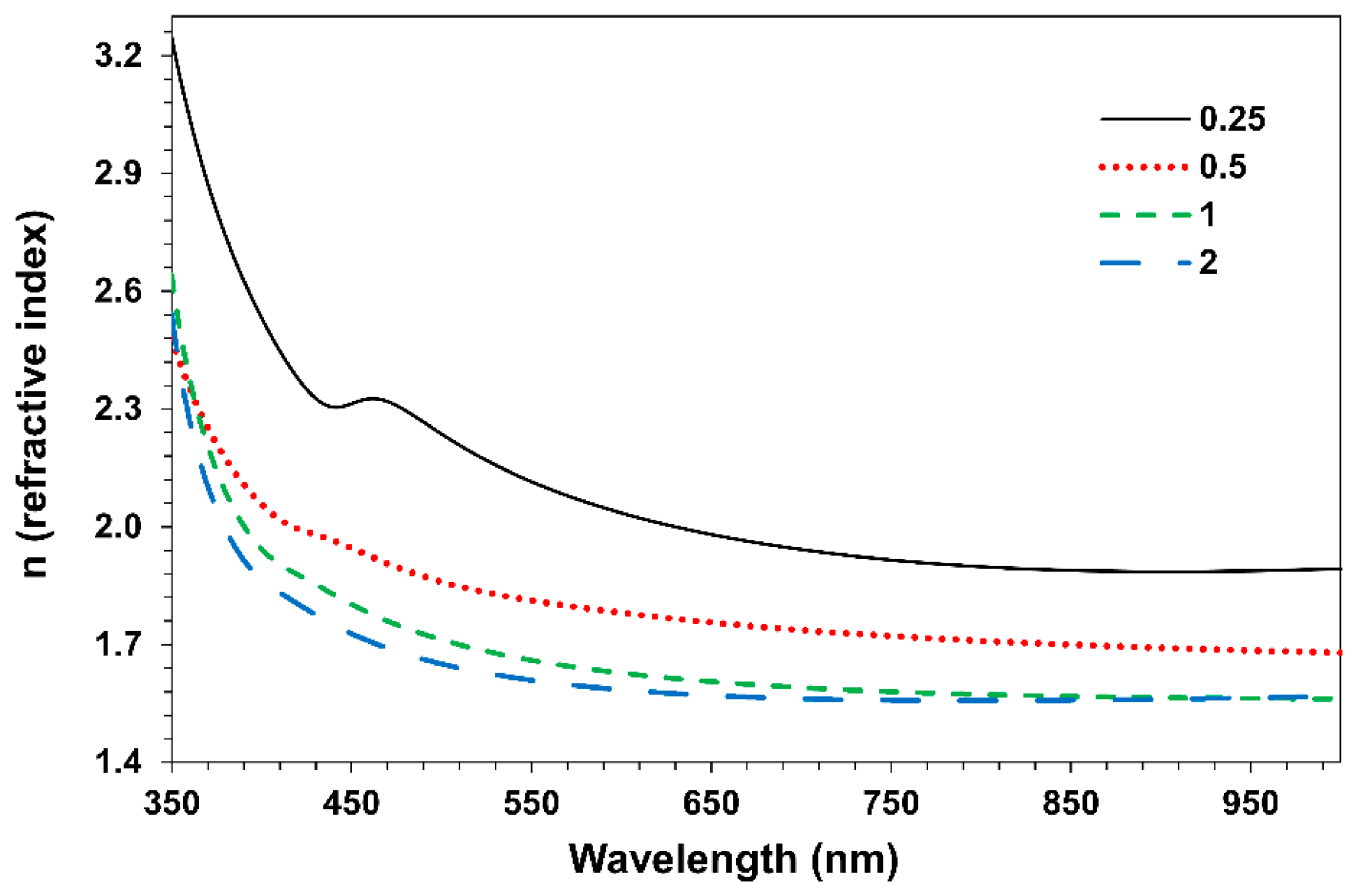
Wavelength-dependent refractive index of MPS-capped CdS quantum dot thin films prepared at different MPS:Cd molar ratios.

Figure 4 shows the wavelength-dependent variation in the refractive index of the films. In addition, the refractive index values of the films at 550 nm are listed in Table 1. The refractive index values at 550 nm range from 2.12 to 1.61, lying below the refractive index of bulk crystalline CdS, approximately 2.60 at 550 nm [22], and above that of SiO_2_, approximately 1.46 at 550 nm [23]. As clearly seen in Figure 4, the refractive index of the films decreases with increasing MPS ratio. In the present system, the SiO_2_ network is formed through the trimethoxysilane groups of MPS, while the thiol groups interact with CdS. Therefore, the decrease in refractive index with increasing MPS ratio can be mainly attributed to the increasing contribution of the MPS-derived SiO_2_ network and the corresponding reduction in the relative CdS content. Consistent with the present findings, a previous study reported that the refractive index of CdS–SiO_2_ composite thin films, in which the SiO_2_ matrix was derived from tetraethyl orthosilicate (TEOS), increased from 1.54 to 1.68 at 632 nm as the CdS volume fraction increased from 13.4% to 29.0% [24]. In Koç’s previous study [25], MPS-capped CdS QDs were synthesized at an MPS:Cd molar ratio of 0.25, and an additional SiO_2_ network was introduced via a TEOS-derived SiO_2_ sol. The thin films with an 80% TEOS-derived SiO_2_–20% MPS-capped CdS QD composition were obtained and heat-treated at 150 °C for 15 min under an N_2_ atmosphere after deposition onto glass substrates. At 550 nm, the refractive index was 1.90 for the MPS-capped CdS QD film with MPS:Cd = 0.25, whereas it decreased to 1.51 for the composite film containing 80% TEOS-derived SiO_2_. These results demonstrate that, regardless of whether the SiO_2_ network originates from the silane functionality of MPS itself or is externally introduced via TEOS, an increase in the silica-based matrix contribution leads to a decrease in the refractive index. However, for similar compositions, the higher heat-treatment temperature applied in the present study, 350 °C in air, results in a relatively higher refractive index compared with the value obtained after heat treatment at 150 °C under N_2_. This indicates that, in addition to composition, the heat-treatment temperature and processing atmosphere also play important roles in determining the final refractive index of the films. Importantly, the present results demonstrate that a controllable refractive index variation can be achieved solely by adjusting the MPS ratio, without requiring any additional SiO_2_ precursor or external silica source such as TEOS.

**Figure 5:**
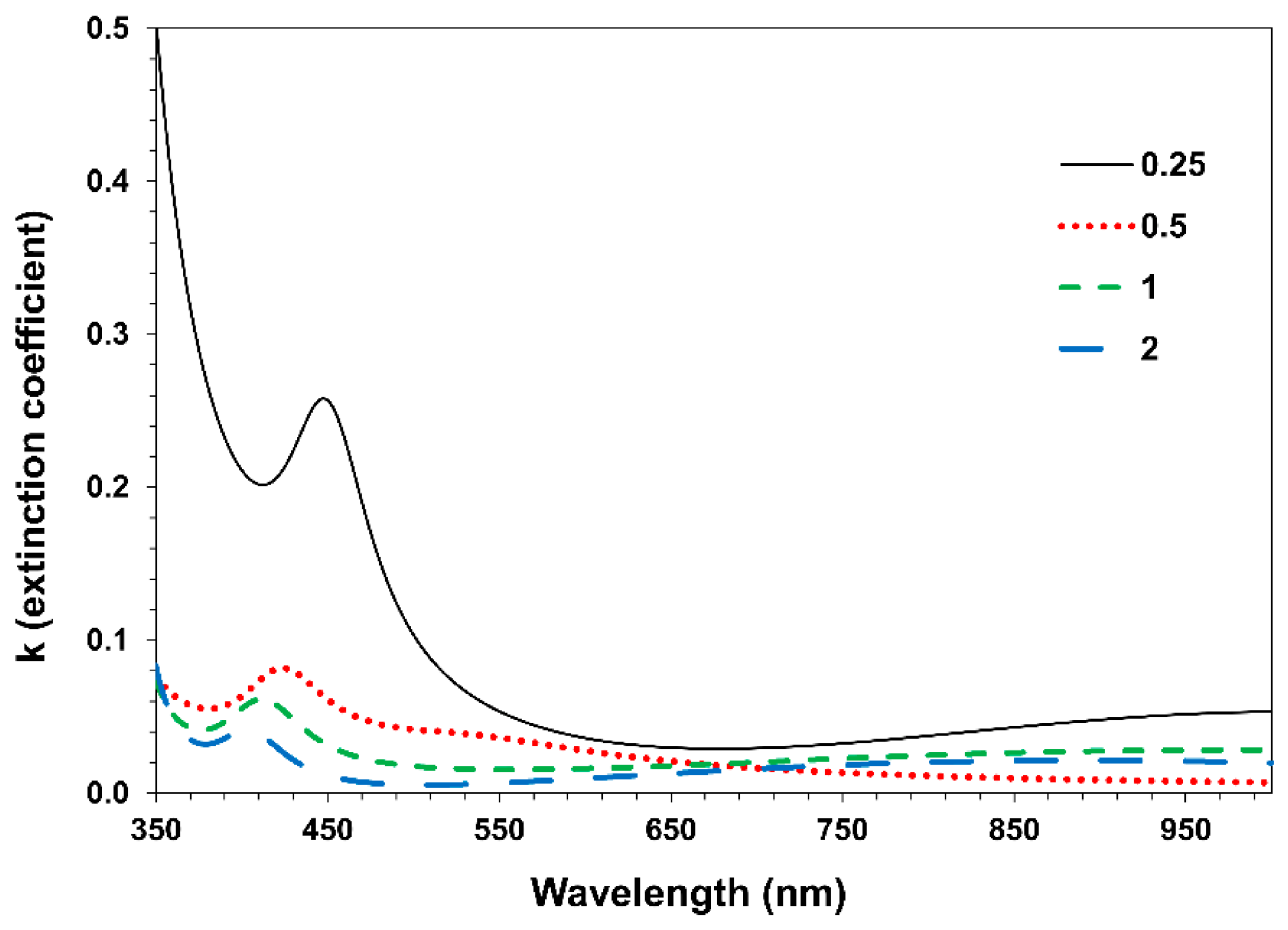
Wavelength-dependent extinction coefficient spectra of MPS-capped CdS quantum dot thin films prepared with different MPS:Cd molar ratios.

Figure 5 presents the wavelength-dependent variation in the extinction coefficient of the films. In addition, the extinction coefficient values at 550 nm are listed in Table 1. As observed in Figure 5, the shoulder in the extinction coefficient spectra shifts toward longer wavelengths with decreasing MPS ratio, which can be associated with changes in the quantum confinement effect. Furthermore, as seen from the spectra, the extinction coefficient decreases with increasing MPS ratio. This behaviour can be explained by the increased contribution of the SiO_2_ network at higher MPS contents, which reduces the relative CdS content and the number of CdS-related absorbing centres in the films. Consequently, the quantum dots become more sparsely distributed within the film matrix as the MPS ratio increases. As a result, the extinction coefficient values decrease with increasing MPS content.

**Figure 6:**
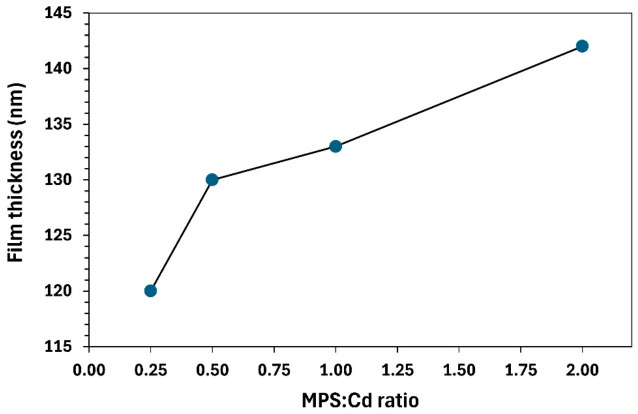
Variation in the film thickness of MPS-capped CdS quantum dot thin films as a function of the MPS:Cd molar ratio.

Figure 6 shows the variation in film thickness as a function of the MPS:Cd molar ratio. As shown in Figure 6, the film thickness increases from 120 to 142 nm as the MPS:Cd molar ratio increases from 0.25 to 2.00. The increase is more pronounced between the samples prepared with MPS:Cd ratios of 0.25 and 0.50, whereas a more gradual variation is observed for the samples with higher MPS contents. This trend may be attributed to the greater contribution of the MPS-derived SiO_2_ network to the overall film structure at higher MPS:Cd ratios. The slower rate of increase at higher MPS contents might also reflect partial surface saturation during deposition or gradual densification of the film. The tunability of film thickness through the MPS:Cd molar ratio is important for controlling the optical response of the films, particularly in thin-film optical applications where both film thickness and refractive index play critical roles.

**Figure 7:**
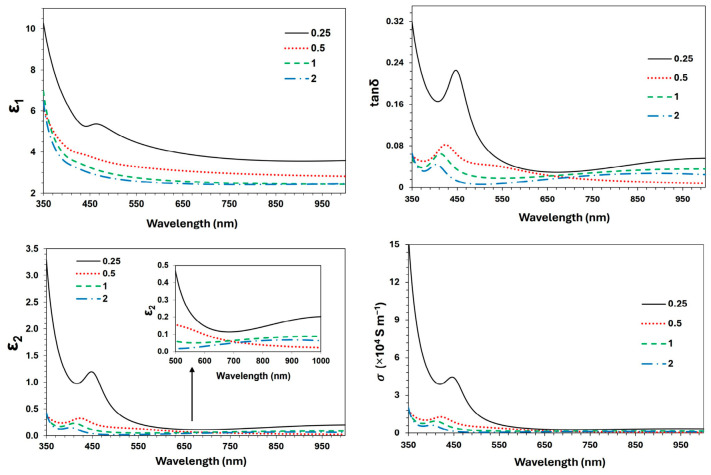
Wavelength-dependent spectra of the real ($\varepsilon_{1}$) and imaginary ($\varepsilon_{2}$) components of the complex dielectric function, the dielectric loss tangent (tan*δ*), and the real part of the optical conductivity (*σ*) for MPS-capped CdS quantum dot thin films prepared with different MPS:Cd molar ratios.

The wavelength-dependent variations in the real $\left(\varepsilon_{1}\right)$ and imaginary $\left(\varepsilon_{2}\right)$ components of the complex dielectric function, dielectric loss tangent (tanδ), and the real part of the optical conductivity $\left(\sigma \right)$ are presented together in Figure 7. These parameters were calculated from the refractive index $\left(n\right)$ and extinction coefficient $\left(k\right)$ to further evaluate the dielectric response and optical loss behavior of the MPS-capped CdS quantum dot thin films.

The real and imaginary components of the complex dielectric function were calculated using the following standard optical relations [26,27]:

(1) $$\varepsilon_{1}=n^{2}-k^{2}$$

and

(2) $$\varepsilon_{2}=2nk$$

respectively, where n is the refractive index and k is the extinction coefficient. The dielectric loss tangent was obtained from the following relation [26,27]:

(3) $$tan\delta =\frac{\varepsilon_{2}}{\varepsilon_{1}}$$

The real part of the optical conductivity, denoted here as σ, was calculated using the following relation [27,28]:

(4) $$\sigma =\omega \varepsilon_{0}\varepsilon_{2}$$

Here, ω is the angular frequency and is expressed as ω=2πc/λ (where c is the speed of light and λ is the wavelength) and $\varepsilon_{0}$ is the vacuum permittivity.

As shown in Figure 7, the film with an MPS:Cd molar ratio of 0.25 exhibits the highest $\varepsilon_{1}$ values, together with a pronounced shoulder around 400–500 nm. This behaviour can be associated with the strong dispersion occurring near the fundamental absorption region of the CdS quantum dots. With increasing MPS content, $\varepsilon_{1}$ values decrease, indicating a reduced polarizable CdS contribution within the hybrid films. Similarly, the imaginary dielectric component $\varepsilon_{2}$ exhibits absorption-related peaks near the fundamental absorption region of the CdS quantum dots and $\varepsilon_{2}$ values generally decrease with increasing MPS content. The observed decreases in both $\varepsilon_{1}$ and $\varepsilon_{2}$ values are related to the increasing contribution of the MPS-derived SiO_2_ network, which becomes more dominant at higher MPS:Cd ratios. On the other hand, the MPS:Cd = 0.25 film shows a slight increase in $\varepsilon_{2}$, becoming more noticeable beyond approximately 750 nm. A similar weak long-wavelength increase is also observed in the extinction coefficient of the MPS:Cd = 0.25 film. Since the photon energies in this spectral range lie below the CdS band-edge absorption region, this weak contribution may be associated with residual sub-band-gap absorption involving deep-level and defect-related states in the CdS-rich film [29]. Light-scattering effects may also enhance the apparent contribution of this already existing sub-band-gap tail response [30].

Figure 7 also shows the dielectric loss tangent, with the highest tan*δ* values observed for the film with an MPS:Cd molar ratio of 0.25, particularly in the near-UV/blue spectral region, where CdS-related optical transitions are more pronounced. It is also noteworthy that, in this spectral region, the tan*δ* values decrease considerably with increasing MPS content. The real part of the optical conductivity also decreases systematically with increasing MPS content over the investigated wavelength range. Since σ depends on both $\varepsilon_{2}$ and the angular frequency, its spectral variation generally follows the trend of the imaginary dielectric component. For quantitative comparison, the optical conductivity values were evaluated at 550 nm. At this wavelength, the σ values were 6861, 3977, 1550, and 644 $S\;m^{-1}$ for MPS:Cd molar ratios of 0.25, 0.50, 1.00, and 2.00, respectively.

When the extinction coefficient is known, the absorption coefficient (*α*) as a function of wavelength can be calculated as [26]:

(5) $$\alpha \left(\lambda \right)=4\pi k/\lambda$$

**Figure 8:**
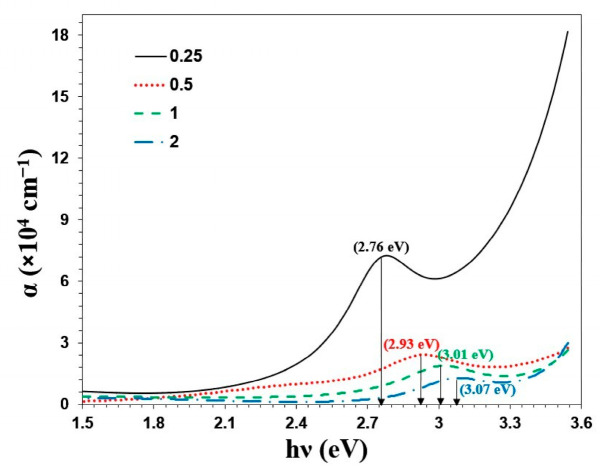
Absorption coefficient spectra as a function of photon energy for MPS-capped CdS quantum dot thin films prepared with different MPS:Cd molar ratios.

The absorption coefficients of the films as a function of photon energy are presented in Figure 8. As can be observed from the spectra, with increasing MPS ratio, the absorption edge (shoulder) shifts toward higher energy (blue shift), which can be attributed to the quantum confinement effect. The first excitonic transition energies $\left(E_{1S-1S}\right),$ determined from the first minima of the second derivatives of the absorption coefficient curves, were found to be 2.76, 2.93, 3.01, and 3.07 eV for the samples prepared with MPS:Cd molar ratios of 0.25, 0.50, 1.00, and 2.00, respectively. The average nanoparticle diameter was estimated from the blue shift in the first excitonic transition energy with respect to the bulk band gap energy ($∆E_{g}$) using the relation given below [31,32].

(6) $$\Delta E_{g}=E_{1S-1S}-E_{g\left(bulk\right)}=\frac{\hbar^{2}\pi^{2}}{2r^{2}}\left(\frac{1}{m_{e}^{*}}+\frac{1}{m_{h}^{*}}\right)-\frac{1.786e^{2}}{\varepsilon r}-0.248\frac{e^{4}}{2\varepsilon^{2}\hbar^{2}}{\left(\frac{1}{m_{e}^{*}}+\frac{1}{m_{h}^{*}}\right)}^{-1}$$

where $E_{g\left(bulk\right)}$ is the band gap energy of the bulk material (2.42 eV for CdS) and r is the radius of the nanoparticle. $m_{e}^{*}$ and $m_{h}^{*}$ are the effective masses of electrons and holes, respectively ($m_{e}^{*}=0.19m_{0}$ and $m_{h}^{*}=0.8m_{0}$ for CdS) and *ε* is the dielectric constant (5.7 for CdS) [31]. The average nanoparticle diameters were estimated by inserting the $E_{1S-1S}$ values into Equation (6), yielding values of 4.13, 3.54, 3.35, and 3.22 nm for the samples prepared with MPS:Cd molar ratios of 0.25, 0.50, 1.00, and 2.00, respectively. These results show good consistency with the previous structural and growth-kinetics study of MPS-capped CdS films by Koç [14], which showed that increasing the MPS:Cd molar ratio suppresses nanoparticle growth during heat treatment. The close agreement between the optically estimated nanoparticle sizes, the systematic blue shift in the excitonic transition energies, and the previously reported structural and kinetic results further supports the reliability of the optical analysis used in this study.

**Figure 9:**
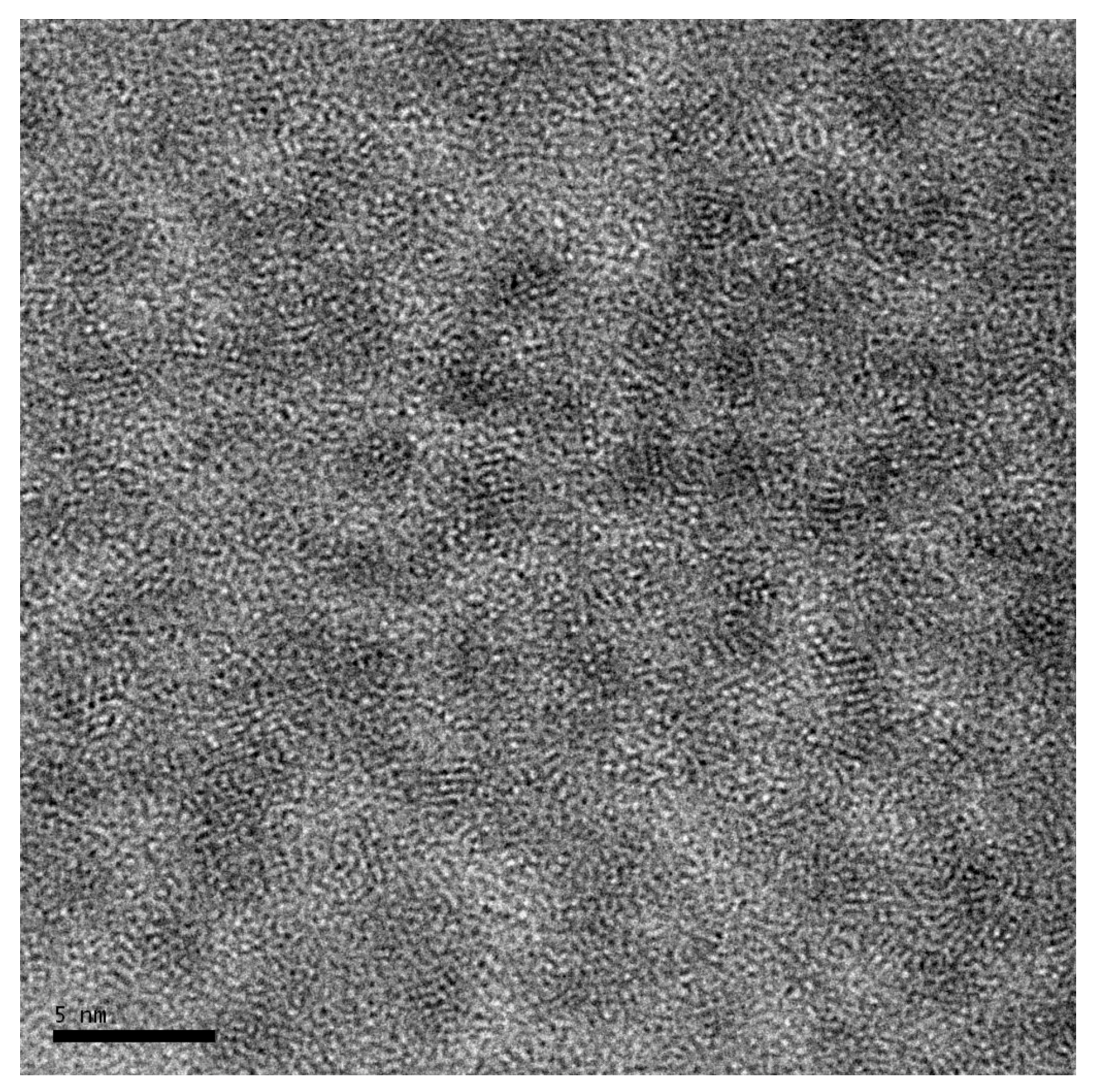
HRTEM image of the unannealed MPS:Cd = 0.25 powder sample. The scale bar represents 5 nm.

An HRTEM image of the unannealed MPS:Cd = 0.25 powder sample is presented in Figure 9. The image indicates an average CdS nanoparticle size of approximately 3 nm. In comparison, the particle size estimated from the $E_{1S-1S}$ excitonic transition energy for the MPS:Cd = 0.25 thin-film sample annealed at 350 °C is approximately 4 nm. This difference is due to particle growth caused by Ostwald ripening during heat treatment [14].

The exponential behaviour observed near the optical absorption edge is generally associated with structural disorder in the material; this region is referred to as the Urbach tail. The Urbach empirical rule is given by the following equation [33,34]:

(7) $$\alpha \left(h\nu \right)=\alpha_{0}\exp\left(\frac{h\nu }{E_{U}}\right)$$

where α is the absorption coefficient, hν is the photon energy, $\alpha_{0}$ is a constant, and $E_{U}$ is the Urbach energy. Taking the natural logarithm yields:

(8) $$\ln\left(\alpha \right)=\ln\left(\alpha_{0}\right)+\frac{h\nu }{E_{U}}$$

**Figure 10:**
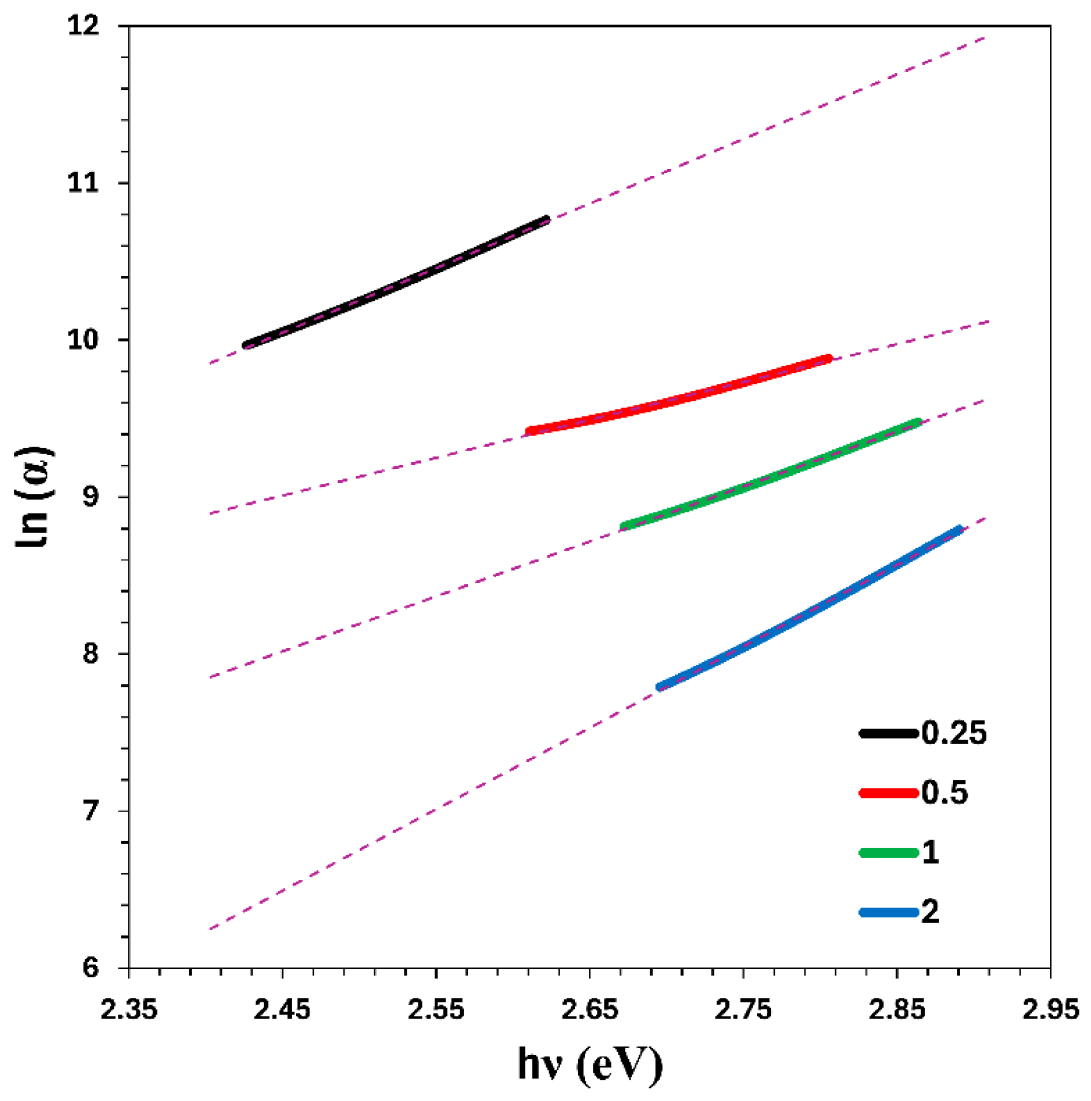
Variation in ln (*α*) with photon energy (*hν*) for MPS-capped CdS quantum dot thin films with different MPS:Cd molar ratios. The dashed lines represent the linear fits used to estimate the Urbach energies.

The Urbach energies $\left(E_{U}\right)$ of the MPS-capped CdS quantum dot thin films were determined from the reciprocals of the slopes of the linear fits to the ln (*α*) vs. *hν* plots shown in Figure 10, according to Equation (8). From these fits, the $E_{U}$ values were found to be 243, 415, 286, and 193 meV for the samples with MPS:Cd molar ratios of 0.25, 0.50, 1.00, and 2.00, respectively.

The obtained Urbach energy values are consistent with previous reports on CdS thin films, as they lie within the energy range reported for cadmium sulfide-based thin-film systems [35,36,37]. The Urbach energy results indicate that the disorder in the MPS-capped CdS quantum dot films does not vary in a simple linear manner with increasing MPS:Cd ratio. Compared with the MPS:Cd = 0.50 sample, the relatively lower Urbach energy at MPS:Cd = 0.25 may be associated with a lower degree of matrix-induced structural disorder due to the limited development of the SiO_2_ network. In contrast, the pronounced increase in Urbach energy at MPS:Cd = 0.50 may be related to enhanced structural competition between the CdS contribution and the MPS-derived SiO_2_ network in this composition. At this intermediate composition, the SiO_2_ network appears to be more developed but has not yet reached a sufficiently continuous structure to provide effective structural homogenization and surface/interface passivation. At MPS:Cd = 1.00, the lower Urbach energy indicates a partial reduction in structural disorder. With a further increase in MPS content, particularly at an MPS:Cd ratio of 2.00, the SiO_2_ network becomes more dominant and more effectively surrounds the CdS nanoparticles. This decrease in Urbach energy can be interpreted as an indication of enhanced structural ordering. Thus, at higher MPS contents, the system not only exhibits higher excitonic transition energy and suppressed particle growth but also evolves toward a more homogeneous and ordered hybrid matrix.

The refractive index data of the MPS-capped CdS quantum dot thin films were analysed using the Wemple–DiDomenico single-oscillator model. The model is expressed as [27,38]:

(9) $$n^{2}-1=\frac{E_{0}E_{d}}{E_{0}^{2}-(h\nu {)}^{2}}$$

where $E_{0}$ is the single oscillator energy and $E_{d}$ is the dispersion energy. For this analysis, the refractive index data were transformed into the linear form of the Wemple–DiDomenico relation:

(10) $$(n^{2}-1{)}^{-1}=\frac{E_{0}}{E_{d}}-\frac{{\left(h\nu \right)}^{2}}{E_{0}E_{d}}$$

**Figure 11:**
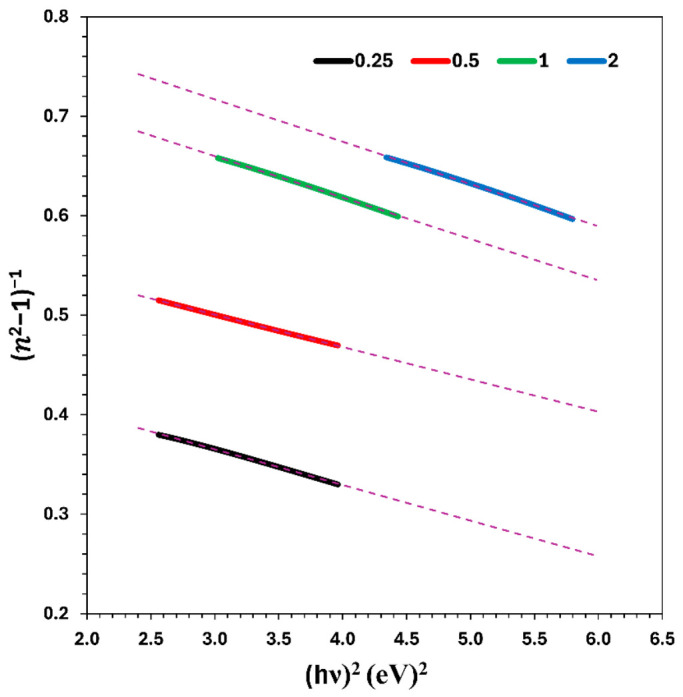
${\left(n^{2}-1\right)}^{-1}$ as a function of ${\left(h\nu \right)}^{2}$ for MPS-capped CdS quantum dot thin films prepared with different MPS:Cd molar ratios. The dashed lines indicate the linear fits used to determine the Wemple–DiDomenico single-oscillator parameters $E_{0}$ and $E_{d}$.

Accordingly, ${\left(n^{2}-1\right)}^{-1}$ was plotted as a function of ${\left(h\nu \right)}^{2}$, as shown in Figure 11, and the $E_{0}$ and $E_{d}$ values were obtained from the corresponding linear fits. The static refractive index $n_{0}$ and the infinite wavelength dielectric constant $\varepsilon_{\infty }$ were calculated using:

(11) $$n_{0}=\sqrt{1+\frac{E_{d}}{E_{0}}}$$

and

(12) $$\varepsilon_{\infty }=n_{0}^{2}$$

The linear susceptibility $\chi^{\left(1\right)}$ was calculated using the following relation:

(13) $$\chi^{\left(1\right)}=\frac{1}{4\pi }(\frac{E_{d}}{E_{0}})$$

The single-oscillator energy $E_{0}$ increased systematically with increasing MPS:Cd ratio, from 3.63 eV for MPS:Cd = 0.25 to 4.29, 4.34, and 4.45 eV for MPS:Cd = 0.50, 1.00, and 2.00, respectively. The higher $E_{0}$ values obtained at higher MPS:Cd ratios are consistent with the upward shift in the $E_{1S-1S}$ energies. The $E_{0}/E_{1S-1S}$ ratios were found to be 1.32, 1.46, 1.44, and 1.45 for MPS:Cd = 0.25, 0.50, 1.00, and 2.00, respectively. In contrast, the dispersion energy $E_{d}$ decreased from 7.68 eV to 7.17, 5.53, and 5.27 eV with increasing MPS:Cd ratio, respectively. The Wemple–DiDomenico analysis further shows that the static refractive index $n_{0}$ and the infinite wavelength dielectric constant $\varepsilon_{\infty }$ decrease with increasing MPS:Cd molar ratio. As the MPS:Cd ratio increases from 0.25 to 0.50, 1.00, and 2.00, $n_{0}$ decreases from 1.77 to 1.64, 1.51, and 1.48, respectively. Similarly, as the MPS:Cd ratio increases from 0.25 to 0.50, 1.00, and 2.00, $\varepsilon_{\infty }$ decreases from 3.12 to 2.67, 2.27, and 2.18, respectively. The linear susceptibility $\chi^{\left(1\right)}$ decreases from 0.168 to 0.133, 0.101, and 0.094 as the MPS:Cd ratio increases from 0.25 to 0.50, 1.00, and 2.00, respectively. These systematic decreases can be attributed to the reduced relative contribution of CdS quantum dots and the increasing dominance of the MPS-derived SiO_2_ network within the hybrid film structure. In line with the studies of Dimitrov and Komatsu [39,40], the increased contribution of the MPS-derived SiO_2_ network may have played a role in reducing the effective electronic polarizability of the film due to the predominance of relatively strong covalent Si–O–Si bonds.

The nonlinear refractive index $n_{2}$ and third-order nonlinear susceptibility $\chi^{\left(3\right)}$ were estimated using the Tichý and Tichá relationship, which is a combination of the generalized Miller’s rule and the static refractive index computed from the Wemple–DiDomenico model [41,42].

The third-order nonlinear susceptibility $\chi^{\left(3\right)}$ was calculated using the following equation:

(14) $$\chi^{\left(3\right)}=A[\chi^{\left(1\right)}{]}^{4}$$

where A is a constant taken to be $1.7\times 10^{-10}$ esu. The nonlinear refractive index $n_{2}$ was subsequently calculated using:

(15) $$n_{2}=\frac{12\pi \chi^{\left(3\right)}}{n_{0}}$$

The calculated nonlinear optical parameters are presented in Table 1. The estimated $\chi^{\left(3\right)}$ values decrease from $1.36\times 10^{-13}$ to $1.34\times 10^{-14}$ esu as the MPS:Cd ratio increases from 0.25 to 2.00. Similarly, $n_{2}$ decreases from $2.91\times 10^{-12}$ to $3.42\times 10^{-13}$ esu. The highest $\chi^{\left(3\right)}$ and $n_{2}$ values obtained in this study are comparable to those reported in the literature for several CdS-based thin-film systems [43]. For $Cd_{0.99}Zn_{0.01}S/ZnO$ nanocomposite thin films, the $\chi^{\left(3\right)}$ and $n_{2}$ values were reported to range from $1.73\times 10^{-13}$ to $1.09\times 10^{-11}$ esu and from $2.6\times 10^{-12}$ to $1.78\times 10^{-10}$ esu, respectively [44]. In another study, the $\chi^{\left(3\right)}$ and $n_{2}$ values for Te-doped CdS thin films were reported to range from $4\times 10^{-13}$ to $3.5\times 10^{-11}$ esu and from $2.4\times 10^{-12}$ to $5.5\times 10^{-10}$ esu, respectively [45]. However, as the MPS content increases, both parameters decrease and progressively deviate from these reported values. This decreasing trend indicates a progressive weakening of the nonlinear optical response with increasing MPS content, which can be attributed to the reduced contribution of relatively more polarizable CdS quantum dots and the increasing dominance of the MPS-derived SiO_2_ network within the hybrid matrix.

## 4. Conclusions

The films prepared with different MPS contents exhibited systematic changes in their optical, dielectric, and nonlinear optical parameters. As the MPS content increased, the film transmittance improved, whereas the refractive index, extinction coefficient, dielectric loss tangent, and real part of the optical conductivity progressively decreased. The Urbach energy did not vary monotonically with increasing MPS content. The highest Urbach energy was obtained for MPS:Cd = 0.50, while the lowest value was observed for MPS:Cd = 2.00. Therefore, the marked increase in MPS content leads to a hybrid matrix with enhanced structural homogeneity and ordering, accompanied by higher excitonic transition energies and restricted CdS particle growth. The blue shift in the first excitonic transition with increasing MPS content is consistent with the Wemple–DiDomenico analysis, which showed that the single-oscillator energy increased from 3.63 to 4.45 eV as the MPS:Cd ratio increased. Moreover, the $E_{0}/E_{1S-1S}$ ratios were confined to a narrow range of 1.32–1.46 across all samples, indicating that the relationship between the single-oscillator energy and the excitonic transition energy was maintained throughout the MPS:Cd series. The same analysis further revealed decreases in the dispersion energy, static refractive index, infinite-wavelength dielectric constant, and linear susceptibility with increasing MPS content. Similarly, the estimated third-order nonlinear susceptibility and nonlinear refractive index were reduced at higher MPS ratios. Overall, these results demonstrate that the MPS:Cd molar ratio is an effective compositional parameter for tailoring the optical and dielectric properties of MPS-capped CdS quantum dot thin films.

## Figures and Tables

**Table 1:** Summary of optical and dielectric parameters, Urbach energies, Wemple–DiDomenico dispersion parameters, and nonlinear optical parameters of MPS-capped CdS quantum dot thin films prepared with different MPS:Cd molar ratios.

MPS:Cd	0.25	0.50	1.00	2.00
n (at λ = 550 nm)	2.12	1.81	1.66	1.61
k (at λ = 550 nm)	0.053	0.036	0.015	0.007
$\varepsilon_{1}$ (at λ = 550 nm)	4.47	3.28	2.76	2.59
$\varepsilon_{2}$ (at λ = 550 nm)	0.23	0.13	0.05	0.02
tanδ (at λ = 550 nm)	0.051	0.040	0.019	0.008
σ $\left(S{\;m}^{-1}\right)$ (at λ = 550 nm)	6861	3977	1550	644
$E_{1S-1S}$ (eV)	2.76	2.93	3.01	3.07
$E_{U}$ (meV)	243	415	286	193
$E_{0}$ (eV)	3.63	4.29	4.34	4.45
$E_{d}\;(\mathrm{eV})$	7.68	7.17	5.53	5.27
$n_{0}$	1.77	1.64	1.51	1.48
$\varepsilon_{\infty }$	3.12	2.67	2.27	2.18
$\chi^{\left(1\right)}$	0.168	0.133	0.101	0.094
$\chi^{\left(3\right)}$ (esu)	1.36 × 10^−13^	5.33 × 10^−14^	1.80 × 10^−14^	1.34 × 10^−14^
$n_{2}$ (esu)	2.91 × 10^−12^	1.23 × 10^−12^	4.49 × 10^−13^	3.42 × 10^−13^

## Data Availability

The original contributions presented in this study are included in the article. Further inquiries can be directed to the corresponding author.
